# Environmental Impacts of Perovskite Solar Cell Materials: Transparency and Reproducibility Gaps, and Reporting Recommendations

**DOI:** 10.1002/gch2.70125

**Published:** 2026-07-31

**Authors:** A. Kamal Kamali, Olga Fuentes, Bertrand Laratte, Guido Sonnemann

**Affiliations:** ^1^ Institute of Molecular Sciences University of Bordeaux Talence France; ^2^ Department of Wood and Forest Sciences Laval University Québec City Quebec Canada; ^3^ Arts Et Métiers Institute of Technology University of Bordeaux Talence France

**Keywords:** chemicals, data, data quality, emerging photovoltaic systems, emerging technologies, renewable energy, reproducibility, transparency

## Abstract

As renewable energy deployment grows and silicon solar cells approach their efficiency limits, perovskite solar cells (PSCs) emerge as a promising next‐generation photovoltaic technology. PSCs environmental impacts are assessed via life cycle assessment (LCA), which depends on the availability of high‐quality life cycle inventories (LCIs). In this study, we systematically identified 101 LCIs related to PSC materials, aiming to recommend the most reliable among them. However, we found that all inventories rely on secondary data and frequently omit critical details such as production scale. We also reproduced reported inventories and found large discrepancies in environmental impacts—sometimes differing by several orders of magnitude across sources. These inconsistencies, coupled with poor documentation, prevented the identification of a single best inventory for any material. Instead, we recommend the use of the most detailed inventories characterized by the highest number of inventory flows as a basis to build more transparent inventories. Our findings demonstrate how gaps in transparency, documentation, and reproducibility in PSC materials inventories impede decision‐making and erode confidence in LCA results. To address these issues, eleven steps are proposed when developing LCIs for emerging materials.

## Graphical abstract

Published life cycle inventories for materials used in perovskite solar cell layers were identified through a systematic review and remodeled. The results revealed large variations in environmental impact estimates for the same materials, including methylammonium iodide and formamidinium iodide. Because limited transparency prevents clear interpretation of these variations, an eleven‐step reporting approach is proposed.

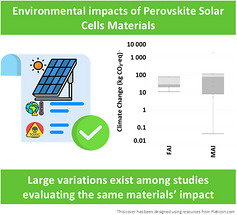

## 1 Introduction

There is no doubt that advancing renewable energy technologies is imperative for sustaining human activities, with wind and solar power leading this transformation. Solar energy, in particular, is experiencing rapid growth; according to the 2024 United Nations Environment Program report, it accounted for over 75% of all new renewable energy installations in 2023 [1]. Currently, the solar energy market is dominated by crystalline silicon (c‐Si) solar cells, which hold a 97% market share in 2023 [2]. However, as c‐Si approaches its intrinsic power conversion efficiency (PCE) limit of approximately 29% [3, 4], the research community is turning to alternative photovoltaic technologies. At the forefront of these emerging technologies are perovskite solar cells (PSCs), which have shown a remarkable increase in laboratory PCE—from 15% in 2015 to a record 26.95% in 2025 [5]. Therefore, PSCs are considered a promising candidate for the future of solar energy, offering strong potential to achieve even higher efficiencies at lower costs [6].

Today, environmental sustainability, alongside economic and performance metrics, is essential in the development of emerging photovoltaics. Life cycle assessment (LCA), a standardized environmental assessment method [7, 8], is increasingly integrated into the research and development of PSC technologies. In this context, LCA aims to support eco‐design decisions and steer the development of environmentally conscious photovoltaics. This proactive application of LCA is expanding, reflected in a growing body of literature examining the environmental implications of various PSC configurations [9]. Several review articles have analyzed these studies [9, 10, 11, 12], discussing the environmental implications and hotspots of PSC technologies and materials, comparing PSC environmental impacts with other emerging and commercial photovoltaics, and highlighting how the modeling choices—such as functional units and system boundaries—influence the results. One notable review attempted to compare findings by harmonizing modelling parameters through mathematical manipulation and excluding studies that did not align with their selected criteria [11]. This approach allows for rapid comparison without requiring access to life cycle inventory (LCI) data. While harmonizing LCA studies is important for identifying environmentally sustainable solutions, the approach used in this case is suboptimal. It relies solely on the reported environmental impacts without reproducing or critically evaluating the quality of the underlying data. Taken together, these reviews fall short of assessing the reliability of the inventories used in LCA models, instead assuming that the data are accurate and representative of real‐world conditions.

High‐quality LCI data are fundamental for drawing reliable conclusions from LCA models. In the domain of emerging technologies, it is common for LCA studies to rely on literature‐based data in constructing inventories due to the unavailability of primary data. This practice has led to several recurring observations, revealed by previous harmonization efforts of LCA studies on emerging carbon fiber production and recycling technologies [13]. For instance, (i) some works repeatedly cite and reproduce the inventory from a single earlier work; (ii) others reference studies that ultimately trace back to the same original source; and (iii) some use data from non‐LCA research without clearly explaining how the inventory was constructed. These practices create confusion and leave readers uncertain about the quality of the inventories—and, by extension, the reliability of the results and conclusions. Addressing these issues requires systematic evaluation of LCA studies on emerging product systems by mapping all published inventories, tracing their origins, assessing their quality, and identifying those that offer high detail and a reasonable representation of real‐world processes.

Here, we undertake such mapping based on the methodology introduced in a recent publication [13] and apply it to PSC, a key technology in the renewable energy transition. We start by conducting a systematic search identifying all available material inventories, including all chemicals, precursors, and solvents used across PSCs’ layers. PSC consists of five layers: front electrode, electron transport layer (ETL), perovskite absorber, hole transport layer (HTL), and back electrode [14]. We make a differentiation between constituting materials embodied in one of PSC five main layers, and those embodied in additional layers or as ancillary materials (e.g., solvents). We then reproduce all identified constituting materials inventories and evaluate their quality based on three criteria: data source, level of detail, and relative environmental impacts. Our aim is to shed light on the degree of data quality of inventories used as a basis for assessing the potential environmental impacts of PSC and support more robust assessments by guiding researchers toward the most reliable and representative inventories available.

## 2 Results and Discussion

Our systematic search has revealed the existence of 98 reproducible inventories for 41 materials used across PSC main layers, additional layers, and as auxiliary materials, as shown in Table 1.

**TABLE 1:** Number of life cycle inventories available for the 41 materials used across perovskite solar cell layers.

PSC Layer	Constituting Materials	Abbreviation	No. of LCIs
Front Electrode	Fluorine doped tin oxide glass	FTO	1
ETL	Titanium dioxide	TiO_2_	7
	Tin dioxide	SnO_2_	1
	Phenyl‐C61‐butyric acid methyl ester	PCBM	4
	Zinc oxides	ZnO_x_	3
	Barium lanthanum tin oxide	Ba_0.95_La_0.05_SnO_3_ – LBSO	1
Perovskite	Methylammonium iodide	CH_3_NH_3_I—MAI	10
	Lead iodide	PbI_2_	9
	5‐ammonium valeric acid iodide	5‐AVAI	5
	Formamidinium iodide	HC(NH_2_)_2_I—FAI	5
	Lead bromide	PbBr_2_	4
	Methylammonium bromide	CH_3_NH_3_Br – MABr	3
	Tin iodide	SnI_2_	1
	Lead chloride	PbCl_2_	2
	Tin bromide	SnBr_2_	1
	Cesium bromide	CsBr	1
	Cesium iodide	CsI	2
HTL	Nickel oxides	NiO_x_	4
	Poly(3,4‐ethylenedioxythiophene) polystyrene sulfonate	PEDOT:PSS	3
	Poly(3‐hexylthiophene)	P3HT	3
	2,2′,7,7′‐Tetrakis(N,N‐di‐p‐methoxyphenylamine)‐9,9′‐spirobifluorene	Spiro‐OMeTAD	2
	Liquid Electrolyte (Lithium iodide)	LiI	1
	Zirconium dioxide	ZrO_2_	1
Back Electrode	Silver	Ag	2
	Carbon	C	1
	Platinum	Pt	1
Additional Layer	Blocking layer‐ Titanium dioxide	BL‐TiO_2_	3
	Titanium oxide nanorods	TIO_2_ Nanorods	1
	Bis‐C60	Bis‐C60	1
Auxiliary Materials			
	Nitrocellulose	(C_6_H_7_O_2_)(NO_2_)_x_	1
	Ethyl cellulose	[C_6_H_7_O_2_(OC_2_H_5_)_3_]_n_	2
	Terpineol	C_10_H_18_O	2
	2‐(2‐butoxyethoxy) ethyl acetate	C_10_H_2_0O_4_	1
	Graphene	G	1
	Graphene quantum dots	GQDs	1
	Lithium bis(trifluoromethanesulfonyl)imide	LiTFSI	2
	Molybdenum disulfide	MoS_2_	1
	Hydrogen bis(trifluoromethanesulfonyl)imide	HNTf_2_	1
	Dimethyl sulfoxide	DMSO	1
	1,8‐Diiodooctane	—	1
	Reduced graphene oxide	rGO	1

**FIGURE 1:**
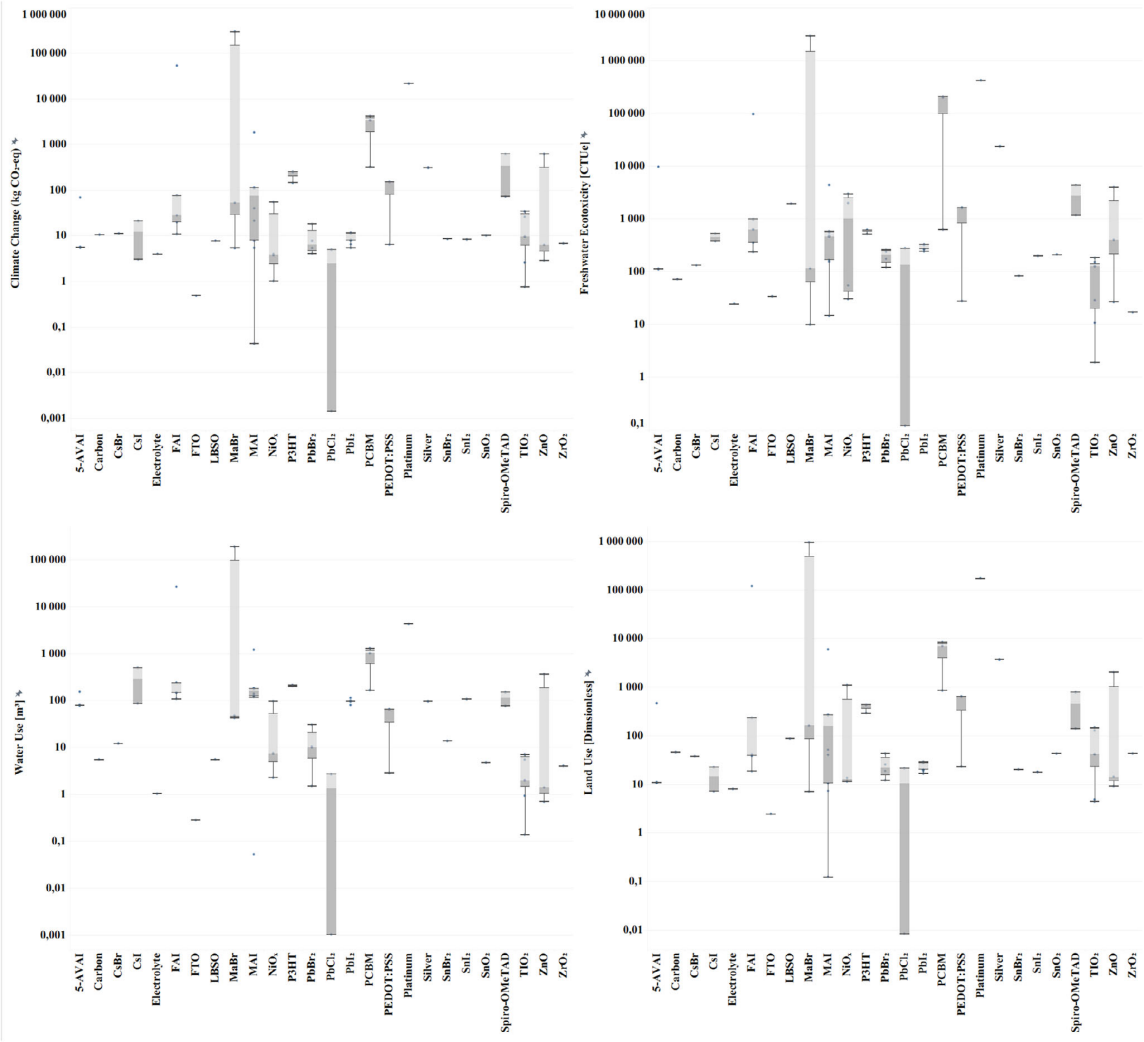
A box and whiskers plot illustrating the climate change impact, freshwater ecotoxicity, human carcinogenic toxicity, and human non‐carcinogenic toxicity for 27 perovskite solar cell materials identified based on the 63 unique inventories selected according to EF 3.1 life cycle impact assessment method.

Initially, 79 inventories were identified for the 27 constituting materials used across the PSC five main layers, see Table 1. However, upon closer examination, 11 of these inventories were found to be duplicates and were therefore not presented in Figure 1. The duplicates are as follows:

- All inventories from Li et al. [15] (covering 5‐AVAI, MAI, P3HT, PbI_2_, PCBM, PEDOT:PSS) were duplicates of those from Li et al. [16].
- The inventories for PbI_2_ from Li et al. [16] and Khalifa et al. [17] were duplicates of Gong et al. [18].
- The inventory for TiO_2_ from Serrano‐Luján et al. [19] duplicated that of Espinosa et al. [20].
- Inventories for 5‐AVAI from Singh et al. [21], and Li et al. [16] were duplicates of Alberola‐Borras et al. [22].

After removing these duplicate entries, 68 distinct inventories remained. These are presented in Figure 1 and discussed in this section. Notably, some inventories were based on previously identified inventories (see Table S3), but the authors made modifications that resulted in different environmental impacts. These modified inventories are included in Figure 1.

### 2.1 Environmental Impacts

Figure 1 presents the environmental impacts of 27 constituting materials embedded in one of the five main layers of PSCs: the front electrode, ETL, perovskite absorber, HTL, and back electrode. These environmental impacts are based on 68 unique inventories and are reported for four impact categories:

- Climate Change Impact (CCI)
- Freshwater Ecotoxicity (FE)
- Water Use (WU)
- Land Use (LU)

These four impact categories aim to adequately represent the environmental challenges most relevant to photovoltaics, spanning climate, ecosystem, and natural resources.

The environmental impacts of all 41 materials, including auxiliary materials and those used in additional layers, across the full set of 16 impact categories are provided in Table S4.

The environmental impacts of 11 of the identified PSC materials (i.e., FTO, Electrolyte, LBSO, SnI_2_, SnBr_2_, SnO_2_, CsBr, Carbon, ZrO2, Bis‐C60, and Platinum) have been reported in only one inventory each, while 16 materials (i.e., MAI, PbI_2_, FAI, 5‐AVAI, TIO_2_, CsI, PCBM, PEDOT:PSS, Spiro‐OMeTAD, MABr, PbCl_2_, PbBr_2_, P3HT, Silver, NiO_x_, and ZnO) are represented by two or more inventories from different research articles. The CCIs of the 11 materials vary significantly, ranging from 0.49 kg CO_2_‐eq for FTO to approximately 22 000 kg CO_2_‐eq for platinum.

At first glance, this variation suggests that PSC materials can have either low or high carbon footprints, substantially influencing the overall environmental impact of PSCs themselves. However, upon closer examination of the environmental impacts resulting from these inventories, we start to question their reliability. For example, we were able to verify whether the replicated CCI values matched the originals for two materials: FTO and LBSO. This was possible because these articles reported the original CCI values per kilogram of material. For FTO, the original research reports 1.33 kg CO_2_‐eq per kg [18], nearly three times higher than our result (0.49 kg CO_2_‐eq). This discrepancy stems from omitting to report the specific ecoinvent datasets used in the original study. In contrast, our calculated CCI for LBSO (7.75 kg CO_2_‐eq) closely aligns with the original value of 7.71 [23] kg CO_2_‐eq.

These comparisons highlight two important points: first, the necessity of transparent and detailed reporting to ensure reproducibility (a topic discussed in the following section), and second, the questionable accuracy of reproduced values for the remaining materials due to insufficient means of verification.

**FIGURE 2:**
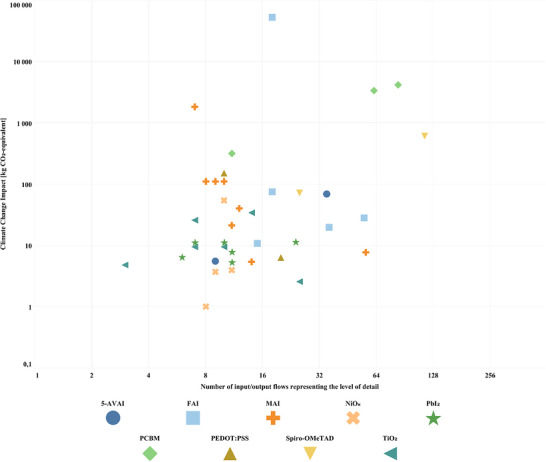
Climate change impacts of nine perovskite solar cell materials based on 36 inventories in relation to the level of detail of their inventories.

Turning to the 16 materials with multiple data sources, we observed substantial variation in reported CCIs across different inventories. A notable example is MAI (Figure 2), a key component of the perovskite absorber layer, for which we found ten different inventories; nine of which are from distinct sources or have been modified by the authors. Reported CCIs for MAI range from as low as 0.04 kg CO_2_‐eq to as high as 1,842 kg CO_2_‐eq per kg of MAI. The lowest value corresponds to an inventory where only 0.01 kg of input materials is claimed to produce 1 kg of output, while the highest value results from an inventory that assumes roughly 5 MWh of electricity consumption to yield just 1 kg of MAI. Within this wide range, three inventories report impacts around 100 kg CO_2_‐eq per kg of MAI, all of which are based on the work of Gong et al. [18].

Similar inconsistencies appear with other materials. For instance, reported CCIs for FAI range from 10 to nearly 54 000 kg CO_2_‐eq per kg (Figure 2). The upper‐end value is based on an inventory that uses tons of raw materials and megawatts of electricity to produce 1 kg of FAI. This may be due to inventories created at the lab scale or possibly due to reporting errors, such as confusing the production of 1 ton with that of 1 kg. These extreme values often come from two specific research articles [23, 24], both of which report unusually large quantities of input materials.

Even when inventories are based on earlier studies, their results often differ from those studies. For example, one inventory reports the CCI of NiOx as 55 kg CO_2_‐eq, while another inventory citing it reports just 1 kg CO_2_‐eq (Figure 2). This discrepancy arises because the original inventory includes 82.8 kg of nickel metal, whereas the dependent inventory mistakenly uses 0.828 kg, likely due to a copying or unit conversion error.

Moreover, large differences also exist between materials inventories dependent on different sources, even in the absence of extreme input/output assumptions. For instance, Spiro‐OMeTAD has CCIs of 73 and 616 kg CO_2_‐eq; TiO_2_ ranges from 2.6 to 34.2 kg CO_2_‐eq; and 5‐AVAI has impacts of 5 and 69 kg CO_2_‐eq per kg (Figure 2). This wide variation in the environmental impacts of the same material across different articles is evident for most PSC materials, as exemplified in Figure 2 for CCI, although the extent of the variation differs.

Although this work sets out to identify the most reliable data, the overall lack of transparency has severely limited the ability to do so. We therefore recommend using the most detailed inventories as a starting point (i.e., those with a high number of inventory flows) for developing more transparent versions that can be independently examined by other researchers (Table S3). To support this, we outline the reporting items that should be communicated. As it stands, most existing LCAs of PSCs are open to criticism due to their reliance on questionable material inventories.

We do not discuss every variation observed across all impact results shown in Figures 1 and 2, as these variations arise from differences in the data used to construct the inventories, particularly the level of detail and the system boundaries. Other potential sources of variation were controlled through remodeling with consistent parameters, including the functional unit, LCIA method, and LCI database. This means that when consistent values do appear, they usually trace back to a shared original source. The overall lack of transparency in inventory construction severely limits further analysis aimed at identifying the original drivers of this variation. A simple explanation of this situation is that these inventories often belong to the background system of perovskite solar cell LCAs, whereas the studies typically focus on detailing the fabrication of the solar cell and the construction of its foreground inventory, rather than on the background system. Therefore, the discussion in the subsequent section focuses on identifying reproducibility constraints rather than variability sources.

### 2.2 Steps for Reproducible Inventories

Focusing on the 18 studies that did include one or more materials inventories (see Table 2), we found the following gaps:

- 1 study (5%) did not report which LCI database was used.
- 9 studies (50%) did not specify the version of the LCI database.
- 10 studies (55%) did not identify the exact datasets used to model the inventory flows.
- All 18 studies (100%) reported the life cycle impact assessment (LCIA) method used.
- 13 studies (72%) did not indicate the version of that method.
- 15 studies (83%) did not report LCIA results for 1 kg of material.

**TABLE 2:** The reporting of the six reproducibility elements within the 18 research articles containing materials inventories.

Refs.	Database	Database version	Datasets reported	LCIA method	LCIA method version	LCIA results for 1 kg of material
[25]	✓	✗	✗	✓	✗	✗
[20]	✓	✓	✓	✓	✗	✗
[19]	✓	✓	✓	✓	✗	✗
[18]	✓	✓	✗	✓	✗	✓
[26]	✓	✓	✓	✓	✗	✗
[27]	✓	✓	✗	✓	✗	✗
[28]	✓	✗	✗	✓	✓	✗
[22]	✓	✗	✗	✓	✓	✗
[24]	✓	✓	✓	✓	✗	✗
[17]	✓	✓	✓	✓	✓	✓
[23]	✓	✗	✗	✓	✗	✓
[29]	✗	✗	✗	✓	✗	✗
[30]	✓	✗	✓	✓	✗	✗
[16]	✓	✗	✗	✓	✗	✗
[31]	✓	✓	✓	✓	✓	✗
[21]	✓	✗	✓	✓	✗	✗
[15]	✓	✗	✗	✓	✗	✗
[32]	✓	✓	✗	✓	✓	✗

As a result, only 6 studies reported all of the following: the materials inventory, the LCI database and its version, the LCIA method, and the datasets used. Notably, in studies that did report the datasets used, this was done inconsistently; the dataset for some flows was documented, while others were not. Among these 6 studies, only 2 reported the version of the LCIA method used. On the other hand, just 3 studies provided LCIA results for 1 kg of each PSC constituent material for which an inventory was built. Only one study reported all six elements necessary for full reproduction of results [17]. Reporting LCIA results in this manner is critical as it allows verification that the reproducible results match the original results.

A reproducible LCA study requires several items to be reported, as identified in a previous research on harmonizing carbon fiber production and recycling technologies [13]. These elements included:

1. The functional unit.
2. The reference flow.
3. The LCIA method.
4. The LCI database.
5. The full dataset's name and geography.

We expand this list to include:

1. The version of the LCI database.
2. The version of the LCIA method.
3. The LCIA results for 1 kg, 1 g, or 1 unit of each constituent material/component.

During our review of published LCA studies on PSC, aimed at extracting and reproducing material inventories, we found that many of these essential items were often missing. A total of 39 published studies were identified that performed LCA on PSCs. Of these, 87% (34 studies) shared inventory data, but only 46% (18 studies) included detailed materials inventories. This is significant because PSCs rely on advanced, complex, and specialized chemicals that are not represented in standard LCI databases. Therefore, the reproducibility of PSC‐related LCAs depends heavily on the availability and transparency of the materials inventories used.

A transparent LCA study not only requires reporting elements that ensure reproducibility but also a clear and systematic explanation of the modelling choices, such as the selection of the functional unit, definition of system boundaries, and other modelling assumptions. In this research, we observed that the methods used to construct PSC inventories are rarely communicated in detail. While researchers often cite materials science papers describing the synthesis procedures of specific materials, they typically do not explain how these procedures were translated into LCIs. In some cases, references include chemical formulas, laboratory protocols, and patents, but the process of integrating these inputs into a coherent, structured inventory is generally not well documented. In addition to this gap, essential characteristics of inventories are often missing. These include the technology scale (e.g., lab‐scale, pilot‐scale, or industrial‐scale) and material properties relevant to each material inventory. Notably, while LCA practitioners studying PSC configurations almost always report the technology scale and efficiency of the solar cell, they frequently rely on secondary data to model both the solar cell and its constituent materials (Table S2). This creates a disconnect: the materials used in each PSC layer must meet specific property requirements to achieve the reported efficiency. Therefore, for transparent construction of PSC materials inventories that lead to credible LCA conclusions, researchers should report:

1. How the inventories were constructed
2. The technology scale, not only for the solar cell but also for its materials
3. The relevant properties of PSC materials.

These details are essential for aligning material inventories with realistic device performance and ensuring the credibility and transparency of the LCA studies.

## 3 Conclusions

In this work, we conducted a systematic search to gather all available LCIs for the materials that constitute PSCs. Our goal was to support future LCAs in the PSC field by identifying and recommending the most reliable available data. To achieve this, we initially examined each inventory based on three key aspects: the technology scale, material properties, and type of data used. Our intention was to prioritize inventories developed at an industrial scale using primary data sources. However, we found that documentation regarding these aspects was often lacking. This appears to be due to researchers assigning less importance to background materials in their assessments (i.e., those materials that are not directly under investigation but are part of the PSC background system).

As a result, we were compelled to evaluate the quality of these inventories through alternative means. Specifically, we conducted mass balance tests, traced the data sources, assessed the level of detail in each inventory, and reproduced the environmental impact calculations to verify the reported results and compare impacts from different sources. Our findings revealed that all identified inventories were based on secondary data, such as technical publications, patents, and, in some cases, previously published LCAs. Most inventories failed the mass balance test. While some inventories were highly detailed, containing up to 50 process flows, others modelling the same materials included fewer than 10, indicating significant variability in comprehensiveness.

In reproducing the CCI values, we harmonized the inventories by removing contributions from transportation, infrastructure, and deposition‐related processes. This revealed that inventories for the same material only produced similar results when they originated from the same source. Inventories based on different sources often showed major discrepancies in environmental impact, with differences reaching several orders of magnitude in some cases. These inconsistencies have made it difficult for us to confidently recommend a single inventory to be adopted as is. Instead, we recommend using highly detailed inventories (i.e., those with a high number of inventory flows) as a starting point for developing more complete and transparent inventories that can be independently examined by other researchers.

To support this goal, we also outline eleven reporting items for future inventories to improve transparency, enable effective verification and validation of reproductions, and facilitate cumulative progress.

## 4 Experimental

Following the methodology introduced in a recent publication [13], we map published inventories by first defining the investigated product system. Next, we identify PSC LCA publications that report inventories using Scopus, an academic research database (Table S1). We then summarize key information regarding the study's research questions and modeling parameters, as well as the PSC architecture and materials, and screen them to only retain those that provide LCIs of PSC materials (Table S2). Subsequently, we harmonized the goal and scope and remodeled the selected materials inventories. This harmonization aligns the functional units, system boundaries, and the life cycle impact assessment method and database. It also standardizes inventory modeling by using consistent datasets for shared flows.

### 4.1 Investigated Product Systems

The material selection is influenced by the PSC structure, which is categorized as either normal (n‐i‐p) or inverted (p‐i‐n). For example, p‐i‐n architectures allow the use of water‐based p‐type materials directly on the substrate—an option not feasible in n‐i‐p designs due to the perovskite layer's sensitivity to humidity and oxygen [33, 34].

Each PSC layer has a precise role in the process of converting the absorbed light into electricity. Sunlight penetrates the transparent conductive glass substrate as well as the electron transport layer in the case of a conventional n‐i‐p structure to be absorbed by the perovskite layer. Next, the perovskite material efficiently harvests solar photons by generating photoexcited charges. Subsequently, charge separation and transference occur by diffusion of the free electrons through the perovskite layer, thanks to its high charge carrier mobility and extraction by the electron transport layer; at the same time, the free holes are extracted by the hole transport layer. Finally, both the back electrode and the transparent electrode collect the separated charges and transfer them through an external circuit, thus generating electricity [35, 36].

### 4.2 Articles Selection and Life Cycle Inventories Screening

To identify and collect LCI data on the materials of PSCs, a search was conducted in the Scopus database on February 24, 2025. The following keywords were used: “life cycle assessment” OR “life cycle analysis” OR “environmental impact*” AND “perovskite solar cell*”. This search returned a total of 203 publications, which are listed in Table S1.

Out of the 203 publications, 57 studies performed an LCA related to PSCs. 18 were excluded for the following reasons: focus on tandem solar cells [37, 38, 39, 40], PSC recycling [41, 42, 43], specific aspects of PSC production (e.g. solvents, electrodes [44, 45], perovskite inks [46], etc. [47, 48].), reproduction of previous LCAs [49, 50], or inadequate description of the LCA method.

Of the remaining 39 studies, 35 studies reported LCI data. Of these, 16 studies were excluded because they provided inventories only for entire PSC configurations rather than for individual materials. The remaining 18 studies, which reported LCIs for specific materials (e.g., chemicals and solvents) used in PSC fabrication, were included. These yielded a total of 101 individual materials inventories, listed in Table S3. These inventories cover a wide range of materials, which are used across the various layers of PSCs. Table 1 categorizes the number of LCIs available for each material by its corresponding PSC layer.

The 101 materials LCIs were selected based on a screening process that excluded inventories reporting input/output flows solely in terms of surface area or volume, without accompanying data to convert these to mass‐based units. Such inventories were excluded because (i) their flows are specific to the deposition methods used and therefore lack generalizability, and (ii) they cannot be harmonized or meaningfully compared due to inconsistencies in reporting formats. As a result, many inventories related to fluorine‐doped tin oxide glass (FTO) have been excluded as their output was expressed in terms of surface area [19, 20, 24]. The only available inventory of indium‐doped tin oxide glass (ITO) was also excluded [18]. Additionally, the materials inventories from Celik et al. (2016) [51] were excluded as they have reported inputs/outputs in units relative to the cell's surface area, without reporting the main product output (i.e., the material) that permits normalization to mass‐based units. Ultimately, only LCIs that provided output data in mass units for PSC constituent materials were retained.

Table S3 summarizes the selected inventories, detailing their data sources, level of detail, and mass balance test results. The data sources help identify the origin of each inventory, checking for shared citations, whether they reference other inventories listed in the table, and whether the sources are based on experimental PSC fabrication or drawn from LCA studies on PSCs or related systems. We have extended the methodology introduced in a past publication on carbon fibers by including [13] (i) the level of detail, measured by the number of input/output flows, indicates how comprehensive each inventory is; and (ii) the mass balance test assesses inventory completeness by verifying whether total inputs match the total outputs. The mass balance test is a well‐established and widely applied method in the LCA field, ensuring that all flows, including dissipative flows [52], are accounted for.

An additional screening process was carried out before reproduction to keep only inventories of constituting materials. Out of 101 inventories, 23 inventories correspond to 11 auxiliary materials, and 3 additional layers were excluded, leaving 78 inventories of 27 PSC constituent materials, as reported in Table 1. During the reproduction process, three inventories could not be reproduced and had to be excluded because they contained material flows not represented in the ecoinvent database. Additionally, the authors did not provide the inventories for those flows or explain how they were modeled. The excluded inventories were: Poly[bis(4‐phenyl)(2,4,6‐trimethylphenyl)amine] (PTAA) [23], Black phosphorus [32], and TNT film [25]. This brings the total number of reproduced inventories down from 101 to 98 for 41 materials.

### 4.3 Goal and Scope Harmonization

#### 4.3.1 The Aim of the Study

This work sets out to investigate the reliability and quality of PSC materials inventories, with the aim of identifying and recommending those that provide the best data available. Initially, we sought to determine the most reliable inventory for each material based on two main criteria: the data sources and the technological scale of production, with preference given to inventories based on primary data at an industrial scale. However, as shown in Table S3, all available inventories rely predominantly on secondary data. Furthermore, while most studies report the technology scale of the solar cell itself (see Table S2), they seldom communicate the production scale of the individual materials. Considering these limitations, we replicated the environmental impact results of all identified PSC materials, as listed in Table 1. For materials with multiple inventories, we graphed their environmental impacts using multiple data points. We then observed the distribution of environmental impacts for each material based on the inventory's level of detail. This permitted us to demonstrate the quality of materials inventories available and used in the LCAs of PSCs, and discuss necessary actions for improving the construction of inventories.

#### 4.3.2 Functional Unit

A mass‐based functional unit of 1 kg is used for each material assessed. This assumes that all inventories for a given material (e.g., FTO) yield functionally equivalent outputs suitable for use in PSCs. This simplification is necessary because existing LCAs do not report detailed material properties relevant to PSC performance.

#### 4.3.3 System Boundaries

A cradle‐to‐gate analysis is conducted, focusing solely on the environmental impacts associated with raw material extraction and production processes of the constituent materials. This excludes the assessment of some of the materials which are synthesized in situ on the substrate, typically by mixing precursor chemicals, such as methylammonium lead iodide and other perovskite material compositions. Only the production of these precursors is included, which we have termed constituent materials. This choice has been made, as synthesizing materials in situ follows laboratory protocols that change from one research to another, and is not in correspondence to our research goal of shedding light on the quality of data used in the background system of assessing PSC configurations.

#### 4.3.4 Life Cycle Impact Assessment Method and Database

The life cycle impact assessment was performed using the environmental footprint (EF) method 3.1, with Cumulative Energy Demand (CED) included as an additional metric. EF [53], an LCIA method regularly updated by the European Commission, bases its toxicity impact categories on the USEtox 2.1 model [54]. USEtox is widely recognized as the most reliable model for assessing toxicity‐related impacts in LCIA due to its broad scientific consensus and updated characterization factors for both chemicals and metals. Among these, lead is a key metal of concern in the development of lead halide PSCs [55]. CED is particularly relevant for evaluating emerging photovoltaic technologies due to its focus on energy use across the life cycle. The model was based on the ecoinvent 3.11 cut‐off database [56], and calculations were carried out in Microsoft Excel.

### 4.4 Life Cycle Inventory Harmonization

#### 4.4.1 Foreground Data Inventory

The foreground system includes all processes and flows necessary for producing the constituent materials of PSCs. This encompasses both material and energy flows, regardless of whether the materials are physically incorporated into the final product. However, it excludes flows related to the deposition method, transportation, and infrastructure. Deposition processes are excluded because they vary across studies, depending on the specific deposition technique and the targeted material composition [57]. Transportation and infrastructure processes are also excluded, as only one study considered transportation [31] and just four addressed infrastructure [15, 16, 22, 26]. Due to this inconsistency in reporting, their exclusion helps ensure a more uniform system boundary across all materials. All input and output flows are linked to background processes using LCI databases, specifically ecoinvent in this study.

#### 4.4.2 Background Data Selection

Background data refers to data points not specific to the investigated product output, often shared across many products and sectors. Examples include water, electricity, and common materials. In this study, background data is sourced from ecoinvent, a leading LCI database. The selection of background processes has been harmonized across the reproduced inventories. Flows of the same nature—such as electricity and tap water—are modelled using the same datasets from the European geography, removing any geographical inconsistencies in the original model. In many cases, inventories did not specify which ecoinvent datasets were used, which undermines reproducibility, see Table 2. For these instances, the most appropriate dataset was selected based on the input/output descriptions and knowledge obtained from modelling inventories that specified the ecoinvent datasets used. The reproduced inventories are reshared along with all ecoinvent datasets used in Table S5, in our effort to allow research continuity and permit data remodeling and refinement.

Proxy datasets were used for five background flows that were not represented in the ecoinvent database and for which no inventory data were provided by the original studies. One article [27] mentioned using a lithium carbonate dataset as a proxy for cesium carbonate; accordingly, in this reproduction, we use the dataset “market for lithium carbonate | GLO.” Another article has used spodumene as a proxy for Pollucite Ore [17]. In this reproduction, we used “market for spodumene | GLO”. The other studies did not specify which proxies were used. Therefore, the following substitutions were made:

- Chloromethane [29]: “market for dichloromethane | RER”
- Hydroxypropyl cellulose [25]: “market for carboxymethyl cellulose, powder,” as an alternative cellulose‐based dispersing agent
- Chloride [18]: “market for sodium chloride, brine solution | GLO”
- Potassium iodate [18, 24]: “market for potassium hydroxide | GLO”
- Liquid hydrogen [15, 16, 21, 22]: “hydrogen production, gaseous, petroleum refinery operation | Europe without Switzerland,” with the reported quantity multiplied by 8.19 (as 1 kg of liquid hydrogen is equivalent to 8.19 kg of hydrogen gas at low pressure)
- Nitrogen gas [25, 27, 31]: “market for nitrogen, liquid | RER” with the reported quantity divided by 1.235 (as 1 kg of liquid nitrogen gives 1.235 kg of nitrogen gas at standard temperature and pressure)
- Hydrogen bromide, emission to water [18]: “Bromide | Emission to water | unspecified”
- Ammonium chloride, emission to water [29]: “Chloride | Emission to water | unspecified”
- Aluminum oxide, emission to water [18]: “Aluminium hydroxide | Emission to water | unspecified”
- Iron chloride, emission to water [20, 24]: “Chloride | Emission to water | unspecified”
- Naphtha, emission to air [31]: “Naphthalene | Emission to air | unspecified”
- Potassium acetate, emission to water [23]: “Acetic acid | Emission to water | unspecified”
- Ethylene glycol monomethyl ether, emission to water [23]: “Ethylene glycol monoethyl ether | Emission to water | unspecified”
- Potassium iodate, emission to air [18, 24]: “Iodine | Emission to air | unspecified.”
- Potassium nitrate, emission to air [18, 23, 24]: “Nitrate | Emission to air | unspecified.”
- Potassium sulfide, emission to air [23]: “Hydrogen sulfide | Emission to air | unspecified.”
- Sodium nitrate, emission to air [20]: “Nitrate | Emission to air | unspecified.”
- 2‐Chloronitrobenzene, emission to air [18]: “Nitrobenzene | Emission to air | unspecified” and “o‐Dichlorobenzene | Emission to air | unspecified”

Given the complexity of the constituent materials used in PSCs, it was often the case that some material flows were not represented in existing LCI databases. In such cases, the authors of the original studies constructed inventories for these missing flows themselves. As a result, many material inventories relied on sub‐inventories that were developed within the same articles. However, some studies did not provide these sub‐inventories or explain how the missing flows were modelled. In our analysis, when such sub‐inventories were available from other studies in our dataset, we used them to fill in the gaps. Specifically:

- Terpineol, used as a component in the LCIs for titanium oxide [18], zirconium oxide [31], and carbon paste [31]: We used the inventory for terpineol developed by Alberola‐Borras et al. [22]
- Potassium iodide, a flow in the LCIs for lead iodide [18, 24] and Spiro‐OMeTAD [18]: We used the potassium iodide inventory from Zhang et al. [25]
- Lead nitrate, used in the LCIs for lead bromide [27, 29]: We used the lead nitrate inventory from Zhang et al. [25]
- Ethyl cellulose, a flow in the carbon paste inventory [31]: We used the inventory developed by Alberola‐Borras et al. [22]

Several anomalies were encountered during the reproduction of the inventories. Some inventories failed to specify the unit for one or more of their inventory flows [17, 19, 25, 31]. In these cases, the missing units were assumed to be in kg. Additionally, one inventory used undefined units such as “KOTA” and “KITM”[34]. These units were also assumed to represent kilograms to ensure consistency. In one instance, a researcher included waste flows of the same material being produced [24]. This was due to the inventory being developed in the context of a specific deposition process—namely, spin coating—where a portion of the deposited material is lost. To maintain a harmonized foreground system, as explained in Section 0, the impacts associated with deposition and annealing processes, along with the waste flows, were excluded from the reproduced inventory. Furthermore, several inventories referenced the ecoinvent dataset for “Electricity, low voltage,” which indicates that the processes were conducted at a laboratory scale [19, 20, 21, 22]. We have maintained the use of this dataset within the reproduced inventories.

## Conflicts of Interest

The authors declare no conflicts of interest.

## Supporting information




**Supporting File**: gch270125‐sup‐0001‐SuppMat.xlsx.

## Data Availability

The data supporting this article have been included as part of the Supplementary Information.
